# Influence Cascades: Entropy-Based Characterization of Behavioral Influence Patterns in Social Media

**DOI:** 10.3390/e23020160

**Published:** 2021-01-28

**Authors:** Chathurani Senevirathna, Chathika Gunaratne, William Rand, Chathura Jayalath, Ivan Garibay

**Affiliations:** 1 Complex Adaptive Systems Lab, Department of Industrial Engineering and Management Systems, University of Central Florida, Orlando, FL 32816, USA; csenevirathna@knights.ucf.edu (C.S.); acj@knights.ucf.edu (C.J.); 2 Computer Science and Artificial Intelligence Lab, Massachusetts Institute of Technology, Cambridge, MA 02139, USA; chathika@mit.edu; 3 Poole School of Management, North Carolina State University, Raleigh, NC 27695, USA; wmrand@ncsu.edu

**Keywords:** influence cascades, transfer entropy, online social networks, cross platforms, cryptocurrency, cyber-vulnerability

## Abstract

Influence cascades are typically analyzed using a single metric approach, i.e., all influence is measured using one number. However, social influence is not monolithic; different users exercise different influences in different ways, and influence is correlated with the user and content-specific attributes. One such attribute could be whether the action is an initiation of a new post, a contribution to a post, or a sharing of an existing post. In this paper, we present a novel method for tracking these influence relationships over time, which we call influence cascades, and present a visualization technique to better understand these cascades. We investigate these influence patterns within and across online social media platforms using empirical data and comparing to a scale-free network as a null model. Our results show that characteristics of influence cascades and patterns of influence are, in fact, affected by the platform and the community of the users.

## 1. Introduction

Social influence in online social networks (OSNs) can be defined as the ability of a user’s action to affect the actions of other users. We refer to such occurrences as social influence relationships. However, in most cases these relationships to be asymmetric. A person who influences other users is referred to as an influencer and the person being influenced is referred to as an influencee. Social influence has been widely studied in many fields including marketing [1,2,3,4,5], political science [6], human and animal behavior [7,8,9,10], and communication [11,12].

With the rapid increase of online social media usage, social platforms now represent a large portion of daily communication and play a major role in information diffusion throughout society. In an OSN, we can classify user actions into three types: (1) initiation of a conversation or a post (I), (2) contribution to an existing conversation or a post (C), or (3) sharing of an existing post between conversations without changing the content (S). Since we will use these three actions and this framework throughout this paper, we will refer to it as the ICS classification. Most existing studies on social influence in OSNs assume an implicit monolithic notion of influence, i.e., that a user’s influence is the same across all action types. However, in reality, there are differences in how users influence others through initiation, contribution, and sharing actions. Disregarding these differences in behavioral influence may hinder a comprehensive understanding of the real role of social influence in a wide variety of scenarios including (1) information propagation and influence maximization, (2) knowledge transfer in a community and development of projects, such as in GitHub and Stack Overflow, (3) online influence campaigns, or (4) online brand engagement at different stages of the consumer purchase funnel.

As an example, in online marketing campaigns, some users may create original content, some users may contribute to others’ created content, and still other users may spread the content of others by sharing. If a marketing firm is interested in controlling or interacting with this information spread, it may want to identify different users based upon the role they play and how those users affect other users. Therefore, in this study, rather than modeling the influence as a single entity, we modeled the influence as multiple entities and explore the cascading effects of social influence.

An influence cascade can be defined as the all of the actions in a chain that start from an initial user, who was prompted by an external (outside the social network) stimulus or intrinsic motivation to act, and the actions that the initial user then influences other users to take, and, in turn, the actions those users influence others to take and so forth until a user’s action no longer influence any other users to act. In other words, an influence cascade is all of the users and events that were socially motivated and can be tracked back to an initial user that was not motivated socially, but due to an influence outside the social network. The presence of influence cascades indicates an underlying organizational structure. In the case of a highly distributed community, such as those that exist on OSNs, such organizational structure is not explicitly expressed, but are implicit in the actions of the users. Analyzing influence cascades allows us to infer these underlying organizational structures. In this paper, we extract influence cascades in a variety of scenarios over multiple platforms and visualize the underlying organizational structures.

Though our introduction of influence cascades is novel, previous work has examined information cascades. However, information cascades differ in that the focus of the analysis is on the transmission of a particular piece of information, and not the users influencing each other to transmit the information. Typically, information cascades are extracted by tracing a piece of information such as content, URL, or an image through the explicit link structures such as parent-child relationships [1,9,13,14,15]. However, such explicit link structures are not available in many data sets or may be incomplete [13,16]. These studies focus on analyzing characteristics such as size, depth, degree distributions, or the growth of such information cascades, as opposed to understanding how one user directly influences another user [1,14,15,17].

As we are interested in behavioral influence, with the premise that any I,C, or *S* action that a user can take can influence other users to do any I,C, or *S* actions, we defined nine types of influence relationships that can exist between any pair of users. We use transfer entropy to quantify these nine types of influence relationships [18] as it gives us the ability to model social influence as multidimensional while capturing the direction and causality of the influence relationships.

Because of the action classification used, our model is abstracted from platform event types and, as a result, we can compare influence cascades on different platforms using the same ICS classification. Different social media platforms enable different affordances for interaction. Though the actions on these platforms can still be characterized under the ICS classification, the algorithms and exact implementations may alter how users utilize these different actions. Hence, this gives us the ability to study human behavior on different platforms and determine if the affordances of the platform affect influence cascades. To the best of our knowledge, this is the first study to compare influence cascades between platforms. We consider cryptocurrency (crypto), common vulnerable exposure (CVE), interest communities on GitHub (GH) and Twitter (TW) to explore the characteristics of influence cascades, and contrast the extracted OSN influence cascades against those from on scale-free networks as well. The results of our study show that the depth and the structure of influence cascades depend on the platform and community of users. As a result of these observations, we can characterize the underlying organizational structures of these online communities.

The rest of the article is organized as follows: Section 2 covers background information and related work. Section 3 presents the proposed methodology, description of empirical data, and the experimental setup. Section 4 present the experimental results on empirical data and a generic scale-free network. Finally, Section 5 discuss the results and Section 6 concludes the paper.

## 2. Background and Related Work

Social influence has long been studied in many areas such as information diffusion and influence maximization [8,11], viral marketing [1,2], influential blogger finding [9], health applications [19], spread of opinions and news [7,12,20], and so on. In these studies, social influence is measured in many different ways. Among these methods, most of the work has focused on the notion of centrality, or structural influence. Centrality measurements such as degree, closeness, betweenness, eigenvector, Katz, and their variations are used widely in studies of social influence [21,22,23,24]. However, in most of these measurements except eigenvector centrality, there is no distinction of the contribution of individual nodes to the measurement [25], and even in the case of eigenvector centrality, the only difference is a structural difference, not behavior-based. The number of followers, which is related to degree centrality, is used by [2,26] to measure influence in microblogs. However, in [2,27,28], the authors show that there is a weak correlation between behavioral influence and the number of followers. Hence, these measurements are not fully able to capture behavioral influence and state-of-art methods related to these measurements cannot comprehensibly address the scenarios where an organization is interested in different types of influence, as discussed in the Introduction.

In addition, some recent studies use deep learning models to capture social influence. The DeepInf developed by Qiu et al. [29] is able to predict the binary status (active/inactive) of a user, given the user’s underlying local network structure and the status of the near neighbors of the user. Leung et al. [30] proposed the HPPNP model by integrating a feature from a page rank domain to the DeepInf model and improved the performance of the DeepInf model. These models use historical interactions to predict social influence. However, the accuracy of the prediction depends on the underlying social network that the model uses because of the assumption that only near neighbors influence users’ actions. In [29,30], the authors use underlying user networks such as follower/followee or friendship networks for their study. Hence, these studies fail to address users’ actions that may occur when they identified posts using hashtags or keywords [31].

Another way to measure influence is based on entropy and information theory. Peng et al. [32] use node entropy based upon the degree of a user and interaction frequency entropy to evaluate social influence in mobile social networks. Sun and Ng [33] use graph entropy based upon the centrality of users to measure the influence of connectors on social networks. Chen et al. [34] consider network topology and proposed a method to rank the influential nodes by considering the Tsallis entropy of the users and their neighbors. Transfer entropy is another entropy-based measurement that is used to quantify influence. Transfer entropy is introduced by Schreiber [18] to capture the cause and effect in an interaction between two coupled systems effectively. It is an information-theoretic approach based on Shannon entropy [35] and it measures the uncertainty reduced by the prediction of the future of a system from the past of the system by knowing the past of another system. If two random processes are $X={\left\{X_{t}\right\}}_{t\in N}$ and $Y={\left\{Y_{t}\right\}}_{t\in N}$ then the transfer entropy can be defined as

(1) $$TE_{X\to Y}=\sum_{x,y\in \Omega }P\left(Y_{t+1}=y,Y_{t}=y,X_{t}=x\right)\log\frac{P(Y_{t+1}=y|Y_{t}=y,X_{t}=x)}{P(Y_{t+1}=y|Y_{t}=y)},$$

where Ω is the sample space that includes all realizations.

VerSteeg and Galstyan [36] use this approach to quantify the influence of content on users in social media and show that transfer entropy is able to capture some of the relationships that cannot be captured by the follower network or mention network successfully. Moreover, He et al. [37] use the same approach to reconstruct the underlying network structure of online social media and use transfer entropy to measure peer influence in OSNs.

Information cascades have provided us with insight into how these social networks operate. For example, Adar and Zhang [13] study the sharing of URLs in the blog-space by inferring their explicit link structure and implicit link structure. Explicit link structure is constructed by tracing the provided information on the data. Implicit link structure is constructed by using a classifier that depends on the blog similarity measures. Gruhl et al. [38] propose a model to study the propagation of information in the form of topics throughout the blog space using a derived form of the independent cascade model on a network induced by the timeseris of the topics and the blog which posts that topic at that time. Further, Leskovec et al. [14] study the propagation of posts in the blog space to discover the patterns of information propagation. The authors analyze the cascades of blog posts by measuring the overall out-degree, in-degree, and in-degree distribution of nodes at level L of the collection of cascades. Further, they quantify the cascades by the number of nodes in the cascades and analyze the distribution of the cascade size over the collection of cascades they extracted. Their results show that blog posts have weekly periodicity but they do not have a bursty behavior. Moreover, Leskovec et al. [1] trace the diffusion of product recommendations using emails and show that product recommendation cascades do not grow very large. Kumar et al. [15] study the information cascades in yahoo!, Twitter, and Usenet groups by reconstructing the information cascades using the parent-child relationships that exist in the data and explore the distributions of size, depth, and degree of the information cascades. They show that degree distributions of information cascades are close to a power law. Bakshy et al. [9] study the information diffusion by studying the cascades of URL’s sharing on Twitter and show that information mainly spreads through small cascades that are started by ordinary individuals while long cascades are rare. Dow et al. [17] study the cascade of image sharing on Facebook and explore them in terms of evaluation time and the distributions of the depth of the cascades. Further, they quantify the predictability of sub-cascades sizes. Cadena et al. [39] show that activity cascades in Twitter are predictive of civil unrest.

Moreover, with the variety of OSNs today, people engage with multiple social media platforms giving them the opportunity to discuss and share their interests in multiple platforms. Hence, researchers have become interested in studying how human behavior differs on different platforms. Xiong et al. [40] propose a new approach to link GitHub and Stack Overflow accounts using a CART decision tree and explore developer behavior on these two platforms. Waterloo et al. [41] study how users express their emotions in WhatsApp, Facebook, Twitter, and Instagram and find that there are differences in the patterns of emotional expression based on the platform. Furthermore, Kim et al. [42] propose a method to estimate the information transfer across mainstream news, social networking sites, and blogs using transfer entropy. Also, a similar study from Bhattacharjee [43] analyzes information transfer across social media, in particular Twitter, Reddit, and GitHub. Bhattacharjee uses symbolic transfer entropy to measure the influence from one platform to the other. Our work extends this past work into the realm of influence cascades, so we can understand not only how the same user operates on different platforms, but also whether users on one platform influence users on other platforms.

## 3. Methodology

In this section, we examine the basic concept of influence cascades. In particular, we start by examining users who are not socially influenced themselves but exert influence on others, and how different actions contribute to the accumulation of social influence as it progresses through the network via influencer-influenced relationships. We use the ICS classification in order to replicate our findings across two social media platforms and two different communities. We extract social influence cascades observed in four online user communities: (1) GitHub users working on cryptocurrency, (2) Twitter users discussing cryptocurrency, (3) GitHub users working on cyber-vulnerabilities (CVEs), and (4) Twitter users discussing CVEs. It should be noted that in this study we are not following any retweet chains or reply chains of specific content. Influence cascades are not direct interaction chains, i.e., retweeting chains or reply chains. Instead, influence cascades are observed from looking at the time series of all users in the data set, and observing how likely a user’s particular event causes another event of another user. This means influence cascades do not always begin with an *I* action because a root user’s *I* action may not be the action that influences other users but instead a root user’s *I*, *C*, or *S* actions could all create influence chains. Also, there is a possibility to observe a *C* action influencing an *I* relationship in the cascade since that means that we observed that when a certain user performs contribution events, another user is likely to initiate a new thread.

We performed this 2 × 2 comparison to give us the ability to analyze both platform and subject community differences. Finally, we compared the extracted social influence cascades against those expected on an artificially generated scale-free network. By using this scale-free model as a null model, we provide a basis of a comparison that is independent of any of the intrinsic properties of underlying networks, to compare and contrast our results. Therefore, we can identify what aspects are related to the particular circumstances of the platform and community and what aspects are present in any network.

### 3.1. Defining Influence Relationships

In this study, we built our framework based on ICS classification. We let the set of actions a user can perform be denoted as A={I,C,S}. Once a user, *u*, performs an action, *a*, there is a chance that his action influences another user, *v*, to preform another action, *b*, which we describe as a social influence relationship of type $u_{a}\to v_{b}$, where a,b∈A and u,v are users in the network *W*. Hence, we can define nine influence relationships as follows: $u_{I}\to v_{I}$, $u_{I}\to v_{C}$, $u_{I}\to v_{S}$, $u_{C}\to v_{I}$, $u_{C}\to v_{C}$, $u_{C}\to v_{S}$, $u_{S}\to v_{I}$, $u_{S}\to v_{C}$, and $u_{S}\to v_{S}$. As an example, we can use $u_{I}\to v_{I}$ to symbolize an influence relationship where *u*’s initiation of a conversation influenced the initiation of another conversation by *v*. We use transfer entropy to quantify these influences and infer causal relationships [18]. Transfer entropy has been shown to capture influence better than other commonly used measures such as centrality and number of followers [36]. Also, by using transfer entropy, we are not restricted to limitations in the follower network that may occur if a user is influenced by, but does not follow, another user [2,28,31].

### 3.2. Extraction of Influence Cascades

We first quantify the magnitude of influence for each relationship $u_{a}\to v_{b}$ by calculating the transfer entropy, from a time series of action type *a* of user *u* to the time series of action type *b* of user *v* [44]. Next, we extract influence cascades from the pruned influence network and visualize them as follows.

#### 3.2.1. Constructing the Influence Network

Since each directed user pair $\vec{\left(u,v\right)}$ can have nine types of influence relationships $u_{a}\to v_{b}$, we define the total social influence from user *u* to *v* as a vector ${\vec{\gamma }}_{u,v}$ with the corresponding influence measurement values $\gamma_{uv(ab)}$ as its vector components. If at least one influence relationship exists, i.e., at least one non-zero influence vector component exists from *u* to *v*, then we can say that *u* influences *v*. Accordingly, we define the influence network G(V,E) according to Equations (2) and (3).

(2) $$V=\{\forall \;u,v\in W|\sum_{a,b\;\in A}\gamma_{uv(ab)}>0\},$$

(3) $$E=\{\left\{u,v\right\}|\sum_{a,b\;\in A}\gamma_{uv(ab)}>0\}.$$

It must be noted that *G* is a directed graph. Furthermore, we attribute the influence vector components to edge weights of *G*. In this manner, *W* is pruned of edges that have no social influence from one user to another, forming *G*.

#### 3.2.2. Extracting Influence Cascades

Next, to study the characteristics and reach of the quantified influences, we extract the influence cascades of the users as follows. Externally motivated but not socially influenced users *R* is defined according to Equation (4).

(4) R={u∈V|in−degree(u)=0}

**Figure 1:**
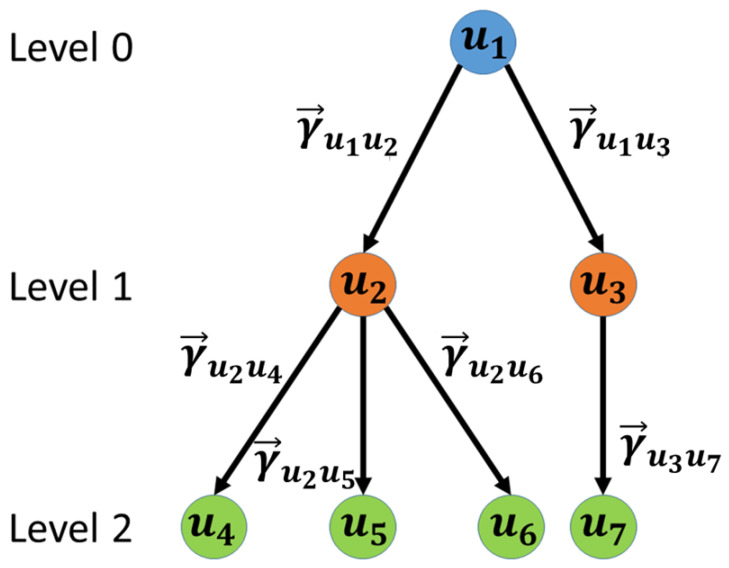
Example of an Influence Cascade. User $u_{1}$ is selected as a root user as it has a zero in-degree, i.e. it is not socially influenced. User $u_{1}$ socially influences users $\left\{u_{2},u_{3}\right\}$ at level 1. Users $\left\{u_{2},u_{3}\right\}$ influence users $\left\{u_{4},u_{5},u_{6},u_{7}\right\}$ at level 2. ${\vec{\gamma }}_{u_{i},u_{j}};i,j=1,2,\cdots ,7$ represent the total influence vector from user $u_{i}$ to user $u_{j}$.

In order to extract the influence cascades from any u∈R, we first extract all the outgoing neighbors of *u*, $N_{o}\left(u\right)$, and their corresponding edges from *u*. We then extract all the outgoing neighbors of users in $N_{o}\left(u\right)$ and their corresponding edges and repeat this process until there are no more identifiable outgoing edges. The initial user, at the top of the cascade, is called the root user and their node level is 0. Level 0 users are chosen as those who have no incoming edges, i.e., have no influencing users, but exert influence on other users. The node level of other users in the cascade is labeled based on the hop distance from the level 0 user to them. Figure 1 shows an example influence cascade using this process.

#### 3.2.3. Characterization of Influence Vector Components

As Figure 1 shows, the extracted cascades help analyze basic characteristics, such as the size and length of the cascades. However, this representation does not identify whether the influencing action was a *I*, *C*, or *S*. Hence, we propose a visualization technique that can integrate the information of influence cascades as follows:

Let $L_{i,i+1}$ represent the set of influence vectors flow from the *i*th level to (i+1)th level, where i∈{0,1,,…,n−1} and *n* is the depth of the cascade. The normalized vector component of the total social influence an action *a* has on an action *b*, $\gamma_{i,i+1(ab)}$ is calculated as shown in Equation (5).

(5) $$\gamma_{i,i+1(ab)}=\frac{\sum_{l\in L_{i,i+1}}l\left(ab\right)}{\sum_{i=0}^{i=n-1}\sum_{l\in L_{i,i+1}}l\left(ab\right)}$$

**Figure 2:**
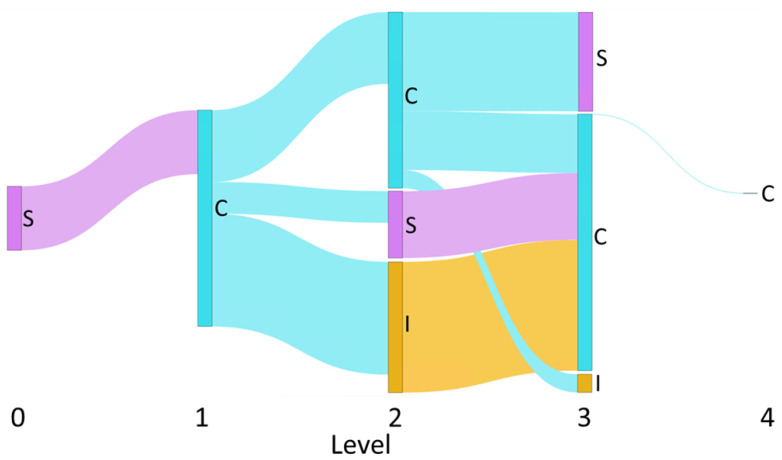
Example of a Sankey diagram produced by the proposed method. The diagram visualizes the normalized flow of total influence, categorized by influence relationships, along the length of a influence cascade. The nodes represent the different activity types, *I*: Initiation (yellow), *C*: Contribution (blue), and *S*: Sharing (pink), and their heights represent the relative magnitude of influence each level exerts on the next. The thickness of the blue, pink, yellow flow lines are proportionate to the magnitude of the normalized total influence value that *C*, *S*, and *I* events have on corresponding actions at the next level, respectively.

We visualize the influence cascade through a Sankey diagram [45]. In the Sankey diagram, nodes represent the influencing actions (a∈A), while flows represent the total magnitude of influence exerted by this action on users at the next level of the cascade, normalized across the cascade. Figure 2 shows an example of a Sankey diagram produced by the proposed method.

### 3.3. Experiments

We studied the consistency of characteristics of influence cascades such as depth of the cascades and the structure of the cascades within and across OSNs. In addition, we compared them against those expected from the scale-free network, which serves as a null model.

#### 3.3.1. Data

We considered two OSNs, Twitter and GitHub for our experiments. Twitter is a popular social networking site that allows users to post and interact with comments. Though GitHub may not appear to be an OSN on its surface, it provides powerful tools for interaction and commenting, allowing users to socially interact in a fashion similar to other OSNs [46].

The empirical data consisted of temporal user activity related to discussions and project development of selected cryptocurrencies (Crypto) and cyber-vulnerabilities (CVE) on both Twitter (TW) and GitHub (GH). The data is gathered as follows:

- GH-Crypto data was collected by extracting events related to more than 20 target coins’ official repositories, repositories labeled with target coin names, and repositories that mentioned the target coin names in their descriptions.
- TW-Crypto data was collected by extracting all tweets from official websites related to more than 20 target coins and by matching the target coin names, code, hashtags, etc. with the full Twitter firehose. Extraction was limited to English language tweets and users from either unknown countries or the UK, India, Canada, Russia, and The Netherlands.
- GH-CVE data was collected by extracting events related to any repositories that were related to CVE at a certain point in their life cycle, found by matching CVE textual patterns against repository descriptions and texts related to events.
- TW-CVE data was collected by matching the CVE textual patterns against the collection of tweets extracted through the public Twitter API.

The TW-Crypto data was extracted from the 1st of August 2018 to the 30th of November 2018 while GH-Crypto, GH-CVE, and TW-CVE data were extracted from the 1st of January 2017 to the 31st of March 2017. The raw data sets contained 111821, 19166, 11875, and 3278 unique users respectively. As low activity users have less impact on influencing others over time we only considered active users who had an average monthly activity greater than five events within these time periods. The filtered data sets contained 4170, 1784, 1989, and 92 unique users respectively. Table 1 shows the categorizations of 14 different GitHub events and 4 different Twitter events into initiation, contribution, and sharing action classes.

The extracted influence networks of GH-Crypto, TW-Crypto, GH-CVE, and TW-CVE had 1406, 3365, 151, and 80 nodes (users), respectively. Each of these influence networks consisted of 568, 2385, 111, and 45 users who were not socially influenced but influenced others (root nodes).

#### 3.3.2. Experimental Setup

**Figure 3:**
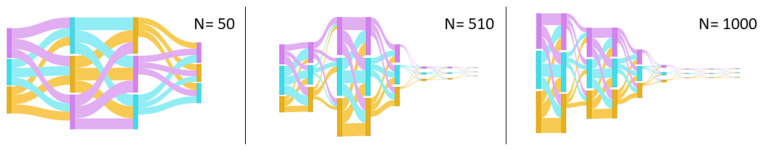
Examples of uniformly distributed influence cascades over scale-free networks of varying network sizes.

We began our experiment by exploring the influence cascades in our empirical networks. For comparison, we constructed generic scale-free networks that were similar in size as null models. As an example, we constructed a scale-free network with 1406 nodes as a null model of GH-Crypto network which has 1406 users in its influence network. Python 3 and the NetworkX scale_free_graph library [47] were used to generate directed scale-free networks. Except for the number of nodes, the other parameter values were kept constant while producing the scale-free networks. Any loops and multi-edges that resulted were removed. Next, for each resultant edge, nine random values from U[0,1] were assigned as the magnitude of the influence of the nine influence relationships. Given this network, we extracted the influence cascades from root nodes by identifying those nodes that had zero in-degree, i.e., no influencing nodes. For each network, we aligned all the influence cascades by level and aggregated the normalized total influence vector components (Equation 5) by their median. For some examples of these cascades see Figure 3.

We explored the user distribution of influenced cascades by comparing the mean number of users as well as the cumulative mean number of users per cascade level by platform and community. The Jensen–Shannon (JS) Divergence test was performed to measure the similarity of user distributions between influence cascades extracted from empirical networks and their null models as well as between the platforms/communities.

In order to study the similarities of the structure of influence cascades in terms of the distribution of influence from different extracted networks, we explored the residual differences between the median normalized total influence values extracted from influence cascades and those from influence cascades generated by the corresponding null model, both within and across platforms, by influence relationship. A Spearman’s correlation test was performed on these residuals by influence relationship, grouping by platform and community, in order to infer the statistical significance of the observations. The null hypothesis $H_{0}$ tested, was that there is no correlation between the residuals in the magnitude of influence of two platform-communities. In other words, if the comparison was significant that means that two platforms or communities are significantly similar in terms of the distribution of the magnitude of influence. As we have multiple comparisons, we applied a Bonferroni correction to minimize the error rate. Therefore, we used a significance level of 0.05/9=0.0055 in order to consider an individual test as significant.

The computer code for extracting influence cascades, visualizations, and experiments was developed in a Jupyter notebook which is publicly available. The influence data extracted from the OSNs and code is available at https://github.com/Csenevirathna/InfluenceCascades. The versions of the software and packages which are used are as follows: Python 3.6.3, pandas 1.0.1, NumPy 1.19.1, NetworkX 2.4, seaborn 0.9.0, Plotly 3.6.0 and, statsmodels 0.12.0.

## 4. Results

**Figure 4:**
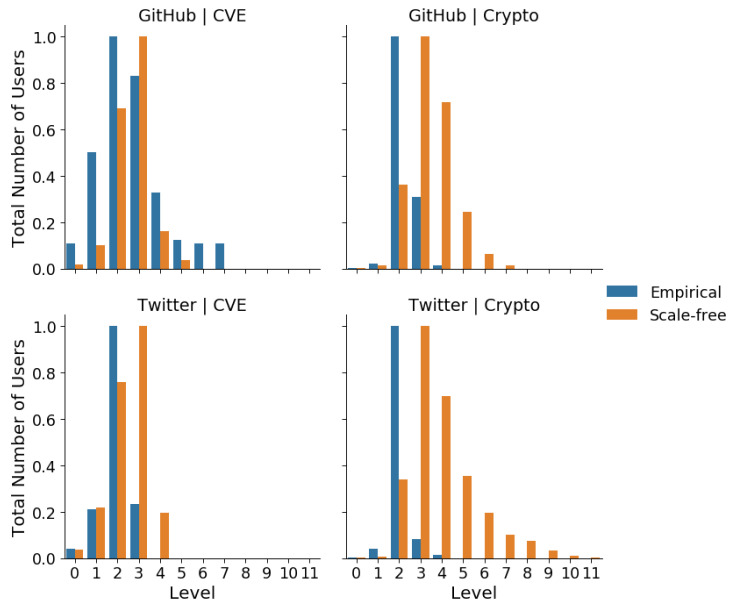
The mean total number of users at each level by platforms and communities for empirical networks and their scale-free networks. The user distribution of the common vulnerable exposure (CVE) community follows the scale-free null model closer than the cryptocurrency community.

**Figure 5:**
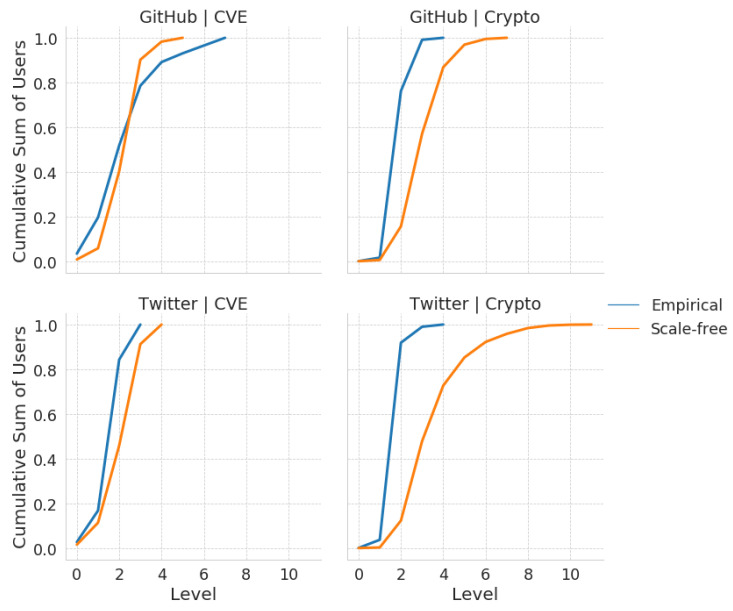
The cumulative sum of the mean total number of users at each level by platforms and communities for empirical networks and their scale-free networks. The cumulative user distribution of CVE community follows the scale-free null model closer than cryptocurrency community.

Comparisons of the mean number of users per influence cascade level by platform and community are shown in Figure 4. Similarly, comparisons of the cumulative mean number of users per cascade level by platforms and community are shown in Figure 5. For both of these sets of measurements, the measurement of the corresponding scale-free null-model, matched by network size, has been included as a control. We observed that the user distributions for the CVE community closely followed that of their corresponding scale-free null-model, in contrast to that of the cryptocurrency community, where a larger deviation from the scale-free null-model was observed. This result is confirmed in Table 2, where the JS-divergences for each platform-community from their corresponding scale-free networks are shown. The JS Divergences for the CVE community networks is a magnitude smaller than that of the cryptocurrency community networks, regardless of platform.

Instead, we found that the distributions of users across levels were similar for the cryptocurrency community, regardless of platform. In particular, we observed that on average the user distributions culminate at level 4 for both cryptocurrency networks, producing influence cascades that are much shorter than are expected based on their comparison against the corresponding scale-free null-models. In other words, the mean user distributions over influence cascades for the cryptocurrency community were robust across platforms, while those for the CVE community were more platform-sensitive. This result is further confirmed in Table 3, where the Jensen–Shannon divergence between each platform-community is displayed. According to this comparison, the JS divergence is lowest within communities rather than within platforms. Furthermore, the JS divergence is lower when comparing across platforms within the cryptocurrency community, rather than the JS divergence when comparing across platforms within the CVE community. Also, we see that the JS divergence between the cryptocurrency and CVE communities on Twitter is much lower than that on GitHub. In other words, the influence structures within the cryptocurrency community are more robust across platforms than the CVE community, and the influence structures on Twitter are more robust across communities in comparison to those on GitHub.

**Figure 6:**
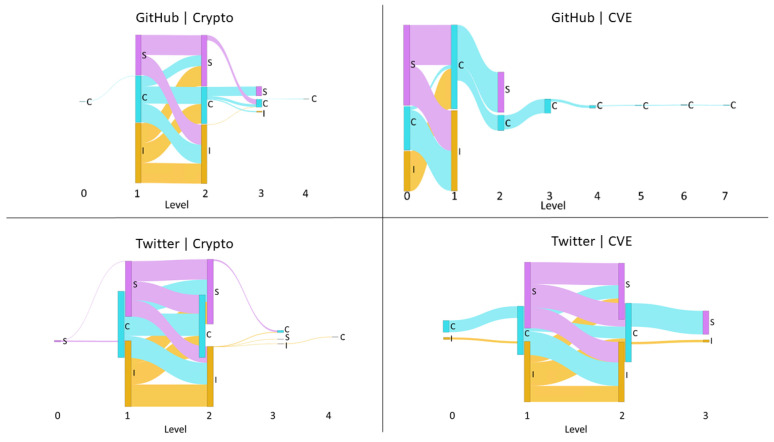
The median normalized total influence, by activity types I,C and *S*, along with the levels of the influence cascades of the GitHub (GH)-Crypto, GH-common vulnerable exposure (CVE), Twitter (TW)-Crypto, and TW-CVE empirical networks. The typical influence cascade in GH-CVE is much longer than the other platform-communities and is dominated by contribution actions influenced by contribution actions.

**Figure 7:**
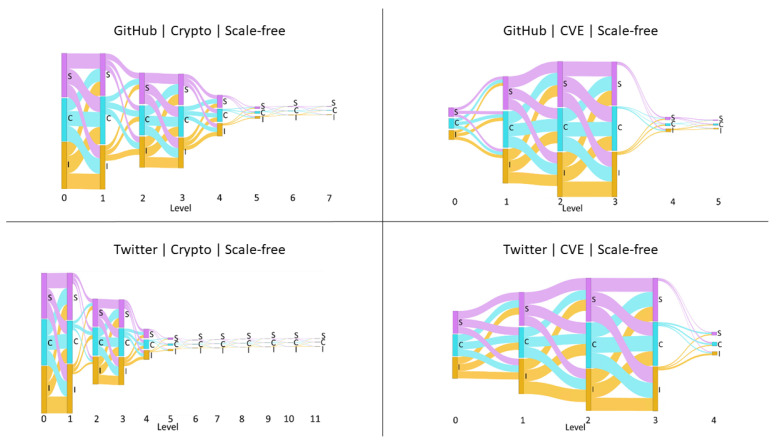
The median normalized total influence, by activity types I,C and *S*, along with the levels of the uniformly distributed influence cascades over the scale-free null models corresponding to GH-Crypto, GH-CVE, TW-Crypto, and TW-CVE influence networks by equal network size.

We then compare the distributions of influence over cascade level by action for the empirical networks against their corresponding scale-free null models matched by network size. Figure 6 displays the median normalized total influence exerted from lower to higher levels by action (I,C, and *S*) for the four empirical networks. The same measurements for their corresponding scale-free null models are shown in Figure 7. Figure 6 shows a clear distinction between how different influence relationships are distributed along the cascades within platforms and across platforms. Despite the closeness of user distributions of the CVE networks to their corresponding scale-free null models, we observe that how influence is distributed among this community differs from that expected through the scale-free null models. We observe a similar difference in influence distribution from the scale-free null models for the cryptocurrency community. We observed that GH-Crypto, TW-Crypto, and TW-CVE have a common shape to their influence cascade, with the highest fraction of influence flow for most relationships in these platform-communities happening towards the middle of the cascade. However, for GH-CVE this happens at the head of the cascade. Interestingly, the distribution of influence seen in GH-CVE, which has a smaller influence network size (151 users), is similar to that seen in the larger networks of GH-Crypto and TW-Crypto scale-free null models (1406 and 3365 users respectively).

These results indicate that root nodes in GH-CVE are more influential compared to all of the other users in the cascade, whereas root nodes in the cryptocurrency community and the TW-CVE community are not very different from the other users in the cascades in terms of the amount of influence they exert on others. This can be explained by the popularity of and interest towards cryptocurrencies among all the users regardless of platform and difference in the interest of users on CVE’s in different platforms. Moreover, these results indicate that influence cascades of empirical networks have less similarity with the scale-free null models by further confirming the effect of communities and platforms on influence cascades.

Furthermore, it was observed that not all nine influence relationships existed between every consecutive level of the influence cascades for any of the social networks, unlike that observed in the scale-free null models. This means that influence exerted by users is not uniform and depends on the type of action they are more inclined to perform given their platform and community, and also that the preference for certain actions is heterogeneous among users of a particular platform and community. Specifically, we observed that influence cascades of Twitter have a more equal distribution of influence through all three actions. Instead, we observe that contribution actions have more influence throughout the cascades observed on GitHub. This result can be explained by the differences in the nature of GitHub and Twitter. That is, GitHub is a platform for developers that are intensively involved in open source software development, but Twitter serves as a platform to share and post short discussions.

**Figure 8:**
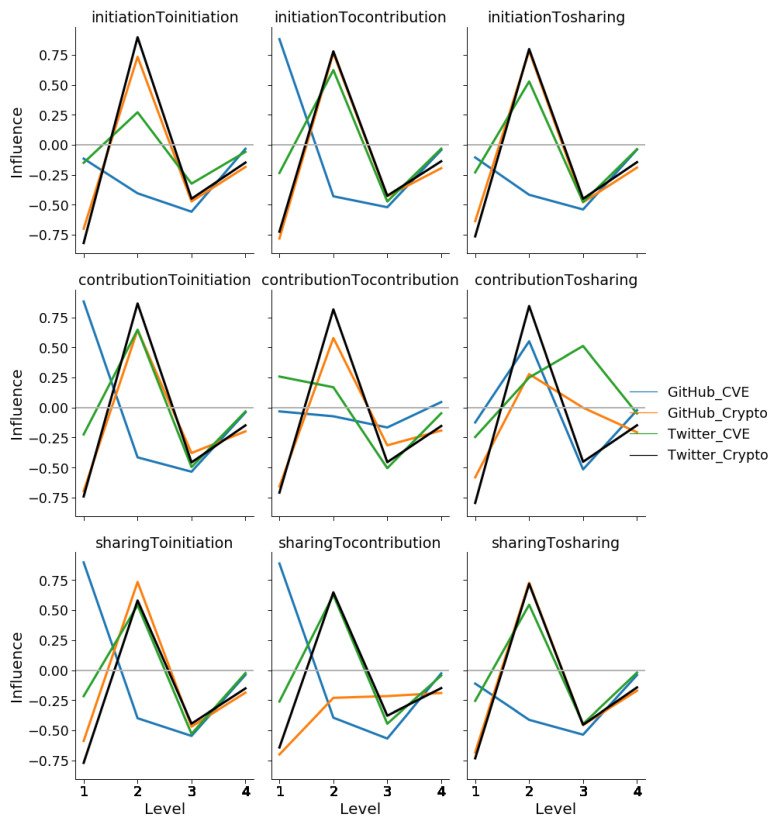
The residuals between the median normalized total influence values by relationship, over cascade level, of the empirical networks when compared to those of their corresponding scale-free null models. Most relationships in the cryptocurrency community seem correlated despite the difference in platform.

The residuals between the median normalized total influence values by relationship, over cascade level, of the empirical networks when compared to those of their corresponding scale-free null models across both platforms and communities are shown in Figure 8. Again we observe that the distribution of influence on GH-CVE is very different compared to that of the other three networks for almost all relationship types, except for C→S. Furthermore, we see that the differences between cryptocurrency community influence cascades on GitHub and Twitter occur through S→C and C→S relationships.

In the case of Spearman’s correlation tests, the null hypothesis for our experiments is that there is no correlation between the residuals in the magnitude of influence of two platform-communities when examined by the nine action-action relationships. The results of this test at original significance = 0.05 (Bonferroni-corrected significance = 0.0055) are shown in Table 4. The only significant correlations were observed between GitHub and Twitter within the cryptocurrency community for most influence relationship types, with the exception of C→S and S→C. In other words, how influence was propagating within the cryptocurrency community over both Twitter and GitHub were similar with the exception of contribution and sharing events. This result can be explained by the higher importance that contribution events (such as commits, and commit comments) have within GitHub, compared against the popularity that sharing (or retweeting) has on Twitter. In contrast, we can state that how influence is propagated within the CVE community differs based on the platform, Twitter or GitHub, upon which the users interact. We can also state that there are no similarities in how influence is propagated when comparing between the two communities on either platform. Appendix A.1 provides further visual validation via scatter graphs for each test above. It must be noted that an ANOVA could not be applied in place of the above correlation test as the residuals of the influence relationships failed to satisfy the normality assumption (Further information in Appendix A.2).

## 5. Discussion

We examine social networks through the perspective of influence propagation based on user actions, and compare four social networks, the cryptocurrency and CVE communities of Twitter and GitHub. In order to facilitate cross-platform comparison, we categorized actions into three abstract types that existed on multiple social media platforms: initiation, contribution, and sharing. The influence of these actions by users on further actions by other users was measured for all nine resulting relationships. We propose a novel method to measure and visualize the social influence exerted by users through these actions over time. We illustrate how transfer entropy can be used as the measurement of influence to estimate the degree to which causal relationships existed between user actions. User pairs that had at least one influence relationship of non-zero magnitude formed the basis of a network of influence. Users of this network that were not influenced by others, but did exert influence on others were selected as the roots of influence cascades. The users influenced by these root influencers were identified recursively, extracting cascades of influence, propagated via all nine relationships. The extracted empirical influence cascades were compared against uniform influence cascades on scale-free networks of equal node count, as null models.

Our results indicate that the manner in which influence cascades through online social media is affected by the social media platform and the online community. In particular, we find that the cryptocurrency community exhibits influence structures that are similar across both GitHub and Twitter, while this is not true for the CVE interest community. More specifically, within the cryptocurrency community, we notice that the only significant difference in influence cascades exist between relationships where contribution actions influence sharing actions, and vice versa. In other words, the influence relationships that exist between users engaged in cryptocurrency related development on GitHub and the influence relationships that exist between users engaged in cryptocurrency discussions are similar, with the exception of contribution actions influencing sharing actions (or sharing influencing contribution). The fact that code-development on GitHub is driven primarily through contribution actions, such as commits and pull-requests, while Twitter is driven by sharing actions, specifically retweets, offers an explanation for this exception. This technique and visualization enable the automatic identification and analysis of these differences.

In contrast, there is no similarity between the influence relationships on GitHub and Twitter within the CVE community. Additionally, we see that the influence cascades of the CVE developers on GitHub are longer than those of the CVE discussions on Twitter. This leads us to conclude that CVE developers on GitHub are generally more responsive to social influence than users discussing CVE related topics on Twitter. Further, we observe generally longer cascades of contribution actions influencing contributions actions within the CVE community on GitHub. In other words, individuals of the CVE community are more likely to engage in contributions to GitHub projects in the CVE domain than engage in CVE related discussions on Twitter.

Finally, we find evidence that the influence structures of Twitter show higher similarity across communities, compared to those of GitHub. However, we do not find any individual influence relationships across the two communities on Twitter that show significantly similar progressions of the magnitude of influence over cascade level.

Some of these differences in platforms versus communities may have to do with the nature of the communities themselves. Cryptocurrencies have been a growing topic since Bitcoin was introduced to the financial market as a medium of exchange. Hence, we could explain the similar organizational structure in the Crypto community as a fact of the popularity of the cryptocurrencies in both Twitter and GitHub. However, the structural differences in the relationships where contribution actions influence sharing actions and sharing actions influence contribution actions can be explained as a result of the different nature of the contribution and sharing actions in GitHub and Twitter.

However, unlike cryptocurrencies, discussions of cyber-vulnerabilities maybe very different on Twitter and GitHub. On Twitter, CVE discussions may be interesting to one group of users who are interested in the news around CVEs, while on Github, the most active users may be individuals who are actively trying to develop solutions to CVEs. This disparity between the types of users engaged on Twitter versus Github is greater for the CVE community than the Cryptocurrency community.

## 6. Conclusions and Future Work

We present one of the first general methods of tracking influence cascades, as opposed to information cascades, on social media, by using a platform-independent action classification and measuring the transfer entropy between timeseries of these actions. We extend the existing literature by discarding the traditional monolithic notion of influence, and by providing new insights into the differences and similarities of how social influence propagates within and across different communities and platforms. Overall, our study contributes to the literature and science by (1) presenting a novel method to track influence relationships caused by actions of OSNs, (2) providing new insights to improve state-of-art methods that assume a monolithic notion of influence and homogeneous populations, (3) characterizing influence cascades caused by actions of social network media across platforms and communities and, (4) presenting the evidence to show that depth and structure of influence cascades are determined by the platform and community.

Although in this study we have analyzed networks within the confines of a specific platform and community, this technique does not limit us from analyzing cross-platform influence cascades. For example, this could happen if a user on GitHub reads a Tweet critical of a CVE, but decides to respond to it not by tweeting, but by modifying or commenting on code pertaining to the respective vulnerability on GitHub instead. Accordingly, future work will extend the current analysis by exploring the characteristics of such cross-platform influence cascades. Moreover, it is also exciting to explore the influence cascades of other communities and platforms in future work.

## Figures and Tables

**Table 1:** Classification of GitHub and Twitter actions.

	Initiation	Contribution	Sharing
**GitHub**	CreateEvent	CommitCommentEvent, GollumEvent, IssueCommentEvent, IssuesEvent, PullRequestEvent, PullRequestReviewCommentEvent, PushEvent, DeleteEvent	ForkEvent, WatchEvent, MemberEvent, PublicEvent, ReleaseEvent
**Twitter**	Tweet	Reply, Quote	Retweet

**Table 2:** Jensen–Shannon Divergence test statistics for each empirical network from its corresponding scale-free null model. Jensen–Shannon (JS) divergences are the smallest within the CVE communities and their scale-free null models, in comparison to that of the cryptocurrency communities.

Community	Platform	JS-Divergence
CVE	GitHub	0.0964
Crypto	GitHub	0.1765
CVE	Twitter	0.0858
Crypto	Twitter	0.2138

**Table 3:** Jensen–Shannon Divergence test statistics between each empirical network. JS divergences are the least within communities and across varying platforms, in comparison to within platforms across varying communities.

Community 1	Platform 1	Community 2	Platform 2	JS-Divergence
Crypto	GitHub	Crypto	Twitter	0.1634
CVE	GitHub	CVE	Twitter	0.1944
Crypto	Twitter	CVE	Twitter	0.1983
Crypto	GitHub	CVE	GitHub	0.3414

**Table 4:** Spearman’s correlation for the $H_{0}$: there is no correlation between the residuals in magnitude of influence of two platform-communities by relationship, at original significance = 0.05 (Bonferroni-corrected significance = 0.0055). For each platform-community, the results for the influence relationships compared are sorted in descending order of correlation coefficient. The only significant correlations are observed between the influence relationships of the cryptocurrency community on Twitter and GitHub, with the exception of C→S and S→C.

Community 1	Platform 1	Community 2	Platform 2	Influence Relationship	ρ	*p*-Value
Crypto	GitHub	Crypto	Twitter	I→I	1	0
				I→C	1	0
				I→S	1	0
				C→I	1	0
				C→C	1	0
				S→I	1	0
				S→S	1	0
				C→S	0.8	0.2
				S→C	0.4	0.6
CVE	GitHub	CVE	Twitter	I→I	0.4	0.6
				I→S	0.4	0.6
				C→C	0.4	0.6
				S→S	0.4	0.6
				I→C	0.2	0.8
				C→I	0.2	0.8
				S→I	0.2	0.8
				S→C	0.2	0.8
				C→S	−0.2	0.8
Crypto	Twitter	CVE	Twitter	I→I	0.8	0.2
				I→C	0.8	0.2
				I→S	0.8	0.2
				C→I	0.8	0.2
				S→I	0.8	0.2
				S→C	0.8	0.2
				S→S	0.8	0.2
				C→S	0.4	0.6
				C→C	−0.2	0.8
Crypto	GitHub	CVE	GitHub	C→S	0.4	0.6
				I→I	0	1
				I→S	0	1
				C→C	0	1
				S→S	0	1
				I→C	−0.4	0.6
				C→I	−0.4	0.6
				S→I	−0.4	0.6
				S→C	−0.4	0.6

**Figure A1:**
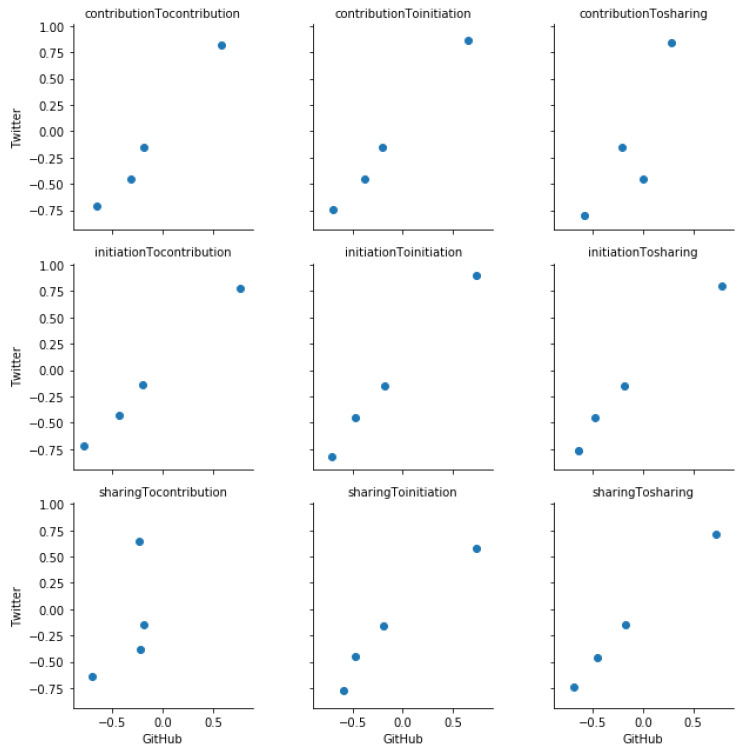
The scatter plot of residuals of median total influence values of GitHub and Twitter platforms in the Crypto community.

**Figure A2:**
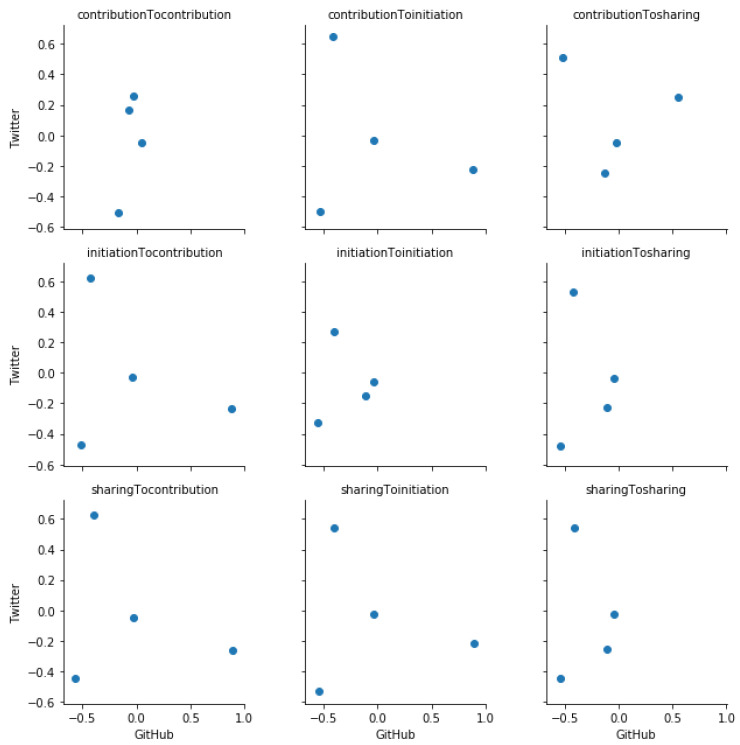
The scatter plot of residuals of median total influence values of GitHub and Twitter platforms in the CVE community.

**Figure A3:**
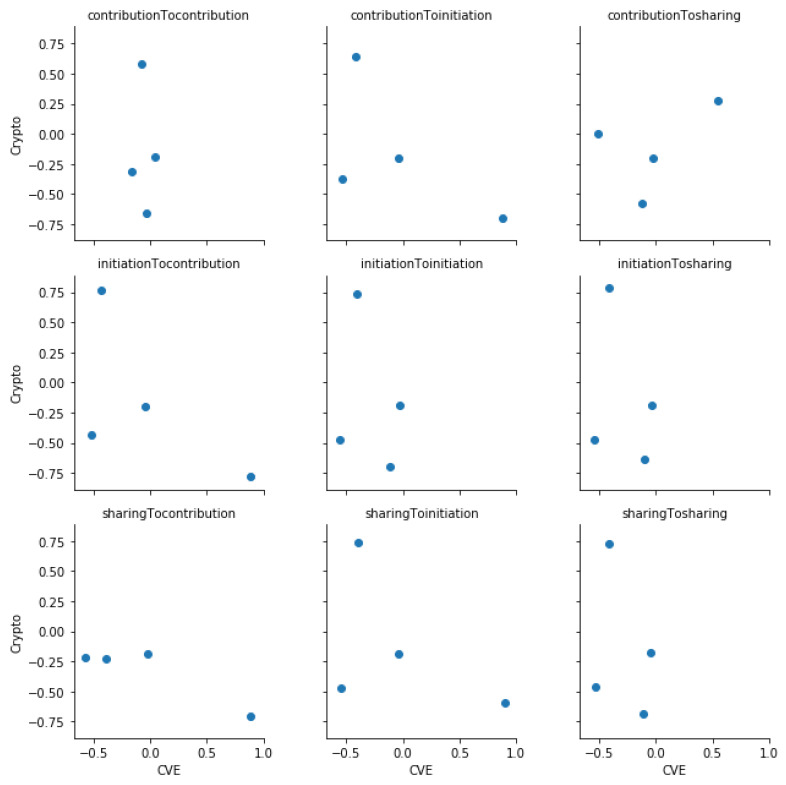
The scatter plot of residuals of median total influence values of the Crypto and CVE community on GitHub.

**Figure A4:**
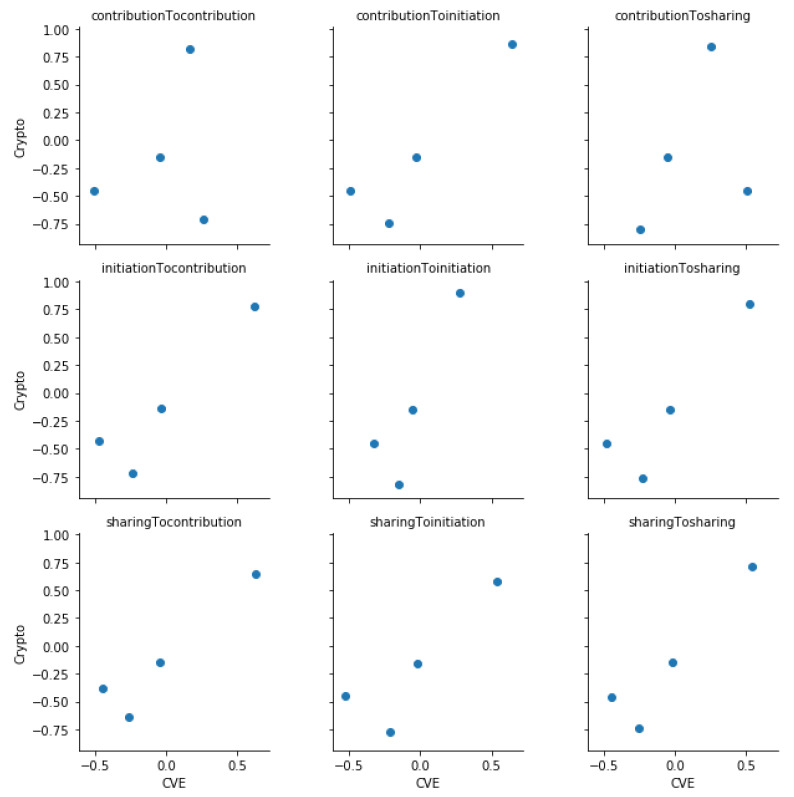
The scatter plot of residuals of median total influence values of the Crypto and CVE community on Twitter.

**Figure A5:**
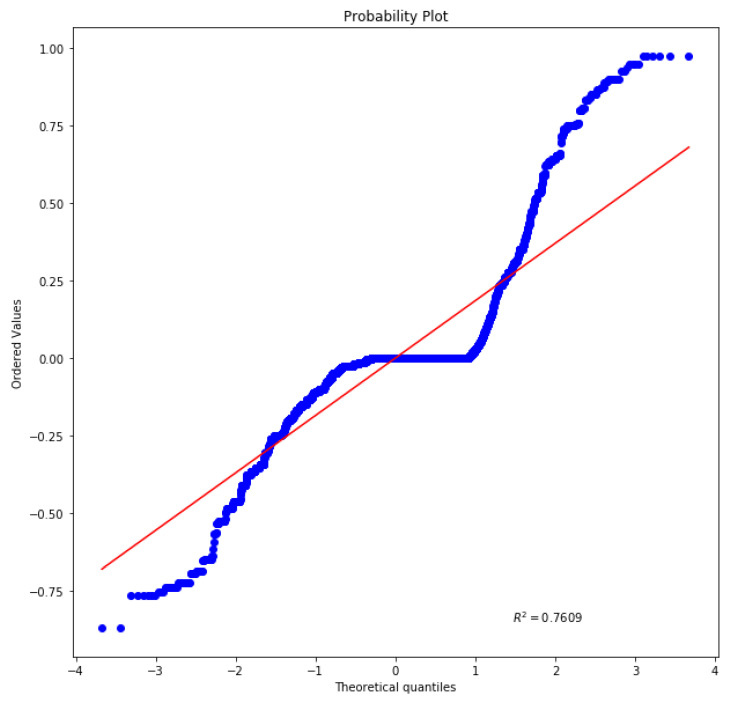
The normal probability plot of residuals of the factorial ANOVA model.

## Data Availability

The data generated and analyzed during this study are openly available in the GitHub repository, https://github.com/Csenevirathna/InfluenceCascades.

## Abbreviations

The following abbreviations are used in this manuscript:

OSN	Online social network
I	Initiation of a conversation or a post
C	Contribution to an existing conversation or a post
S	Sharing of an existing post between conversations without changing the content
Crypto	Cryptocurrencies
CVE	Cyber-vulnerabilities
GH	GitHub
TW	Twitter
JS	Jensen–Shannon

## Appendix A. Statistical Tests

### Appendix A.1. Spearman’S Correlation Test

We present here scatter plots corresponding to each Spearman’s correlation test which we performed. We observe that except for the Crypto community (Figure A1) neither CVE community (Figure A2) nor GitHub (Figure A3), Twitter (Figure A4) platforms have a clear monotonic relation. Further, we observe that contribution to sharing and sharing to contribution influence relationships in the Crypto community do not show a clear monotonic relation.

### Appendix A.2. Factorial ANOVA Test

We examine the use of the factorial ANOVA test as a statistical significance test to infer the similarities of the structure of the influence cascades across platforms and communities. Forty influence cascades are chosen randomly from the set of influence cascades extracted from each empirical influence networks. The residual values between total normalized influence vector components and the median total normalized influence vector components of the influence cascades from the corresponding scale-free network are calculated. Data is grouped by platform-community, influence relationships, and level, and the normality of each data set was examined using the Shapiro-Wilk test. The *p*-values are compared with 0.05. Not all the data sets were able to satisfy the normality assumption as shown in Table A1, Table A2, Table A3 and Table A4. Also, the normality of the residual of the factorial ANOVA model is examined using the Shapiro-Wilk test. However, the test is failed with a *p*-value of less than 0.05. and the Figure A5 shows the nonlinear relationship between theoretical quantities and sample quantities, which support the non-normality of the residuals.

**Table A1:** Test statistics of factorial ANOVA (Part I).

Platform-Community	Influence Relationship	Level	Statistics	*p*-Value	$H_{0}$
GitHub-CVE	contributionTocontribution	1	0.78	2.72 × 10${}^{-6}$	rejected
GitHub-CVE	contributionTocontribution	2	0.9	1.79 × 10${}^{-3}$	rejected
GitHub-CVE	contributionTocontribution	3	0.93	1.53 × 10${}^{-2}$	rejected
GitHub-CVE	contributionTocontribution	4	0.8	8.28 × 10${}^{-6}$	rejected
GitHub-CVE	contributionToinitiation	1	0.39	1.04 × 10${}^{-11}$	rejected
GitHub-CVE	contributionToinitiation	2	1	1.00 × 10${}^{0}$	not rejected
GitHub-CVE	contributionToinitiation	3	1	1.00 × 10${}^{0}$	not rejected
GitHub-CVE	contributionToinitiation	4	1	1.00 × 10${}^{0}$	not rejected
GitHub-CVE	contributionTosharing	1	0.23	3.25 × 10${}^{-13}$	rejected
GitHub-CVE	contributionTosharing	2	0.64	1.05 × 10${}^{-8}$	rejected
GitHub-CVE	contributionTosharing	3	0.61	4.91 × 10${}^{-9}$	rejected
GitHub-CVE	contributionTosharing	4	1	1.00 × 10${}^{0}$	not rejected
GitHub-CVE	initiationTocontribution	1	0.39	1.04 × 10${}^{-11}$	rejected
GitHub-CVE	initiationTocontribution	2	1	1.00 × 10${}^{0}$	not rejected
GitHub-CVE	initiationTocontribution	3	1	1.00 × 10${}^{0}$	not rejected
GitHub-CVE	initiationTocontribution	4	1	1.00 × 10${}^{0}$	not rejected
GitHub-CVE	initiationToinitiation	1	1	1.00 × 10${}^{0}$	not rejected
GitHub-CVE	initiationToinitiation	2	1	1.00 × 10${}^{0}$	not rejected
GitHub-CVE	initiationToinitiation	3	1	1.00 × 10${}^{0}$	not rejected
GitHub-CVE	initiationToinitiation	4	1	1.00 × 10${}^{0}$	not rejected
GitHub-CVE	initiationTosharing	1	1	1.00 × 10${}^{0}$	not rejected
GitHub-CVE	initiationTosharing	2	1	1.00 × 10${}^{0}$	not rejected
GitHub-CVE	initiationTosharing	3	1	1.00 × 10${}^{0}$	not rejected
GitHub-CVE	initiationTosharing	4	1	1.00 × 10${}^{0}$	not rejected
GitHub-CVE	sharingTocontribution	1	0.23	3.25 × 10${}^{-13}$	rejected
GitHub-CVE	sharingTocontribution	2	1	1.00 × 10${}^{0}$	not rejected
GitHub-CVE	sharingTocontribution	3	1	1.00 × 10${}^{0}$	not rejected
GitHub-CVE	sharingTocontribution	4	1	1.00 × 10${}^{0}$	not rejected
GitHub-CVE	sharingToinitiation	1	1	1.00 × 10${}^{0}$	not rejected
GitHub-CVE	sharingToinitiation	2	1	1.00 × 10${}^{0}$	not rejected
GitHub-CVE	sharingToinitiation	3	1	1.00 × 10${}^{0}$	not rejected
GitHub-CVE	sharingToinitiation	4	1	1.00 × 10${}^{0}$	not rejected
GitHub-CVE	sharingTosharing	1	1	1.00 × 10${}^{0}$	not rejected
GitHub-CVE	sharingTosharing	2	1	1.00 × 10${}^{0}$	not rejected
GitHub-CVE	sharingTosharing	3	1	1.00 × 10${}^{0}$	not rejected
GitHub-CVE	sharingTosharing	4	1	1.00 × 10${}^{0}$	not rejected

**Table A2:** Test statistics of factorial ANOVA (Part II).

Platform-Community	Influence Relationship	Level	Statistics	*p*-Value	$H_{0}$
GitHub-Crypto	contributionTocontribution	1	0.73	3.02 × 10${}^{-7}$	rejected
GitHub-Crypto	contributionTocontribution	2	0.89	1.32 × 10${}^{-3}$	rejected
GitHub-Crypto	contributionTocontribution	3	0.91	3.21 × 10${}^{-3}$	rejected
GitHub-Crypto	contributionTocontribution	4	0.3	1.36 × 10${}^{-12}$	rejected
GitHub-Crypto	contributionToinitiation	1	0.42	2.43 × 10${}^{-11}$	rejected
GitHub-Crypto	contributionToinitiation	2	0.77	2.07 × 10${}^{-6}$	rejected
GitHub-Crypto	contributionToinitiation	3	0.77	2.11 × 10${}^{-6}$	rejected
GitHub-Crypto	contributionToinitiation	4	1	1.00 × 10${}^{0}$	not rejected
GitHub-Crypto	contributionTosharing	1	1	1.00 × 10${}^{0}$	not rejected
GitHub-Crypto	contributionTosharing	2	0.95	5.75 × 10${}^{-2}$	not rejected
GitHub-Crypto	contributionTosharing	3	0.95	5.75 × 10${}^{-2}$	not rejected
GitHub-Crypto	contributionTosharing	4	1	1.00 × 10${}^{0}$	not rejected
GitHub-Crypto	initiationTocontribution	1	0.15	6.64 × 10${}^{-14}$	rejected
GitHub-Crypto	initiationTocontribution	2	0.6	3.49 × 10${}^{-9}$	rejected
GitHub-Crypto	initiationTocontribution	3	0.57	1.13 × 10${}^{-9}$	rejected
GitHub-Crypto	initiationTocontribution	4	1	1.00 × 10${}^{0}$	not rejected
GitHub-Crypto	initiationToinitiation	1	1	1.00 × 10${}^{0}$	not rejected
GitHub-Crypto	initiationToinitiation	2	0.63	7.16 × 10${}^{-9}$	rejected
GitHub-Crypto	initiationToinitiation	3	0.23	3.25 × 10${}^{-13}$	rejected
GitHub-Crypto	initiationToinitiation	4	1	1.00 × 10${}^{0}$	not rejected
GitHub-Crypto	initiationTosharing	1	1	1.00 × 10${}^{0}$	not rejected
GitHub-Crypto	initiationTosharing	2	0.6	3.26 × 10${}^{-9}$	rejected
GitHub-Crypto	initiationTosharing	3	0.15	6.64 × 10${}^{-14}$	rejected
GitHub-Crypto	initiationTosharing	4	1	1.00 × 10${}^{0}$	not rejected
GitHub-Crypto	sharingTocontribution	1	0.65	1.72 × 10${}^{-8}$	rejected
GitHub-Crypto	sharingTocontribution	2	0.65	1.68 × 10${}^{-8}$	rejected
GitHub-Crypto	sharingTocontribution	3	0.77	1.70 × 10${}^{-6}$	rejected
GitHub-Crypto	sharingTocontribution	4	1	1.00 × 10${}^{0}$	not rejected
GitHub-Crypto	sharingToinitiation	1	0.15	6.64 × 10${}^{-14}$	rejected
GitHub-Crypto	sharingToinitiation	2	0.29	1.19 × 10${}^{-12}$	rejected
GitHub-Crypto	sharingToinitiation	3	1	1.00 × 10${}^{0}$	not rejected
GitHub-Crypto	sharingToinitiation	4	1	1.00 × 10${}^{0}$	not rejected
GitHub-Crypto	sharingTosharing	1	1	1.00 × 10${}^{0}$	not rejected
GitHub-Crypto	sharingTosharing	2	0.15	6.64 × 10${}^{-14}$	rejected
GitHub-Crypto	sharingTosharing	3	1	1.00 × 10${}^{0}$	not rejected
GitHub-Crypto	sharingTosharing	4	1	1.00 × 10${}^{0}$	not rejected

**Table A3:** Test statistics of factorial ANOVA (Part III).

Platform-Community	Influence Relationship	Level	Statistics	*p*-Value	$H_{0}$
Twitter-CVE	contributionTocontribution	1	0.15	6.64 × 10${}^{-14}$	rejected
Twitter-CVE	contributionTocontribution	2	1	1.00 × 10${}^{0}$	not rejected
Twitter-CVE	contributionTocontribution	3	1	1.00 × 10${}^{0}$	not rejected
Twitter-CVE	contributionTocontribution	4	1	1.00 × 10${}^{0}$	not rejected
Twitter-CVE	contributionToinitiation	1	0.15	6.64 × 10${}^{-14}$	rejected
Twitter-CVE	contributionToinitiation	2	0.62	5.59 × 10${}^{-9}$	rejected
Twitter-CVE	contributionToinitiation	3	0.15	6.64 × 10${}^{-14}$	rejected
Twitter-CVE	contributionToinitiation	4	1	1.00 × 10${}^{0}$	not rejected
Twitter-CVE	contributionTosharing	1	0.22	2.81 × 10${}^{-13}$	rejected
Twitter-CVE	contributionTosharing	2	0.63	7.17 × 10${}^{-9}$	rejected
Twitter-CVE	contributionTosharing	3	0.61	4.53 × 10${}^{-9}$	rejected
Twitter-CVE	contributionTosharing	4	1	1.00 × 10${}^{0}$	not rejected
Twitter-CVE	initiationTocontribution	1	0.59	2.07 × 10${}^{-9}$	rejected
Twitter-CVE	initiationTocontribution	2	0.65	1.67 × 10${}^{-8}$	rejected
Twitter-CVE	initiationTocontribution	3	1	1.00 × 10${}^{0}$	not rejected
Twitter-CVE	initiationTocontribution	4	1	1.00 × 10${}^{0}$	not rejected
Twitter-CVE	initiationToinitiation	1	0.79	4.87 × 10${}^{-6}$	rejected
Twitter-CVE	initiationToinitiation	2	0.85	9.62 × 10${}^{-5}$	rejected
Twitter-CVE	initiationToinitiation	3	0.74	4.97 × 10${}^{-7}$	rejected
Twitter-CVE	initiationToinitiation	4	1	1.00 × 10${}^{0}$	not rejected
Twitter-CVE	initiationTosharing	1	0.62	6.69 × 10${}^{-9}$	rejected
Twitter-CVE	initiationTosharing	2	0.8	7.55 × 10${}^{-6}$	rejected
Twitter-CVE	initiationTosharing	3	0.64	1.21 × 10${}^{-8}$	rejected
Twitter-CVE	initiationTosharing	4	1	1.00 × 10${}^{0}$	not rejected
Twitter-CVE	sharingTocontribution	1	0.15	6.64 × 10${}^{-14}$	rejected
Twitter-CVE	sharingTocontribution	2	0.54	5.09 × 10${}^{-10}$	rejected
Twitter-CVE	sharingTocontribution	3	0.23	3.25 × 10${}^{-13}$	rejected
Twitter-CVE	sharingTocontribution	4	1	1.00 × 10${}^{0}$	not rejected
Twitter-CVE	sharingToinitiation	1	0.45	4.74 × 10${}^{-11}$	rejected
Twitter-CVE	sharingToinitiation	2	0.7	1.17 × 10${}^{-7}$	rejected
Twitter-CVE	sharingToinitiation	3	0.44	3.48 × 10${}^{-11}$	rejected
Twitter-CVE	sharingToinitiation	4	1	1.00 × 10${}^{0}$	not rejected
Twitter-CVE	sharingTosharing	1	0.33	2.45 × 10${}^{-12}$	rejected
Twitter-CVE	sharingTosharing	2	0.71	1.30 × 10${}^{-7}$	rejected
Twitter-CVE	sharingTosharing	3	0.46	6.44 × 10${}^{-11}$	rejected
Twitter-CVE	sharingTosharing	4	1	1.00 × 10${}^{0}$	not rejected

**Table A4:** Test statistics of factorial ANOVA (Part IV).

Platform-Community	Influence Relationship	Level	Statistics	*p*-Value	$H_{0}$
Twitter-Crypto	contributionTocontribution	1	0.36	5.16 × 10${}^{-12}$	rejected
Twitter-Crypto	contributionTocontribution	2	0.65	1.40 × 10${}^{-8}$	rejected
Twitter-Crypto	contributionTocontribution	3	0.56	8.47 × 10${}^{-10}$	rejected
Twitter-Crypto	contributionTocontribution	4	1	1.00 × 10${}^{0}$	not rejected
Twitter-Crypto	contributionToinitiation	1	0.53	3.80 × 10${}^{-10}$	rejected
Twitter-Crypto	contributionToinitiation	2	0.69	7.75 × 10${}^{-8}$	rejected
Twitter-Crypto	contributionToinitiation	3	0.52	2.98 × 10${}^{-10}$	rejected
Twitter-Crypto	contributionToinitiation	4	1	1.00 × 10${}^{0}$	not rejected
Twitter-Crypto	contributionTosharing	1	0.26	6.03 × 10${}^{-13}$	rejected
Twitter-Crypto	contributionTosharing	2	0.61	4.73 × 10${}^{-9}$	rejected
Twitter-Crypto	contributionTosharing	3	0.55	6.01 × 10${}^{-10}$	rejected
Twitter-Crypto	contributionTosharing	4	1	1.00 × 10${}^{0}$	not rejected
Twitter-Crypto	initiationTocontribution	1	0.42	1.96 × 10${}^{-11}$	rejected
Twitter-Crypto	initiationTocontribution	2	0.36	5.14 × 10${}^{-12}$	rejected
Twitter-Crypto	initiationTocontribution	3	0.36	5.42 × 10${}^{-12}$	rejected
Twitter-Crypto	initiationTocontribution	4	0.15	6.64 × 10${}^{-14}$	rejected
Twitter-Crypto	initiationToinitiation	1	0.45	4.32 × 10${}^{-11}$	rejected
Twitter-Crypto	initiationToinitiation	2	0.36	5.45 × 10${}^{-12}$	rejected
Twitter-Crypto	initiationToinitiation	3	0.22	2.70 × 10${}^{-13}$	rejected
Twitter-Crypto	initiationToinitiation	4	0.15	6.64 × 10${}^{-14}$	rejected
Twitter-Crypto	initiationTosharing	1	0.31	1.83 × 10${}^{-12}$	rejected
Twitter-Crypto	initiationTosharing	2	0.25	5.11 × 10${}^{-13}$	rejected
Twitter-Crypto	initiationTosharing	3	0.24	3.90 × 10${}^{-13}$	rejected
Twitter-Crypto	initiationTosharing	4	0.15	6.64 × 10${}^{-14}$	rejected
Twitter-Crypto	sharingTocontribution	1	0.67	2.85 × 10${}^{-8}$	rejected
Twitter-Crypto	sharingTocontribution	2	0.64	1.25 × 10${}^{-8}$	rejected
Twitter-Crypto	sharingTocontribution	3	0.58	1.52 × 10${}^{-9}$	rejected
Twitter-Crypto	sharingTocontribution	4	0.15	6.64 × 10${}^{-14}$	rejected
Twitter-Crypto	sharingToinitiation	1	0.75	7.77 × 10${}^{-7}$	rejected
Twitter-Crypto	sharingToinitiation	2	0.79	4.20 × 10${}^{-6}$	rejected
Twitter-Crypto	sharingToinitiation	3	0.49	1.37 × 10${}^{-10}$	rejected
Twitter-Crypto	sharingToinitiation	4	1	1.00 × 10${}^{0}$	not rejected
Twitter-Crypto	sharingTosharing	1	0.71	1.30 × 10${}^{-7}$	rejected
Twitter-Crypto	sharingTosharing	2	0.58	1.55 × 10${}^{-9}$	rejected
Twitter-Crypto	sharingTosharing	3	0.48	1.06 × 10${}^{-10}$	rejected
Twitter-Crypto	sharingTosharing	4	0.15	6.64 × 10${}^{-14}$	rejected
